# Supplementary material for: Dynalign II: common secondary structure prediction for RNA homologs with domain insertions
Source: Nucleic Acids Res. 2014 Nov 21;42(22):13939–48. doi: 10.1093/nar/gku1172 (PMC4267632; doi:10.1093/nar/gku1172)
Supplement: SUPPLEMENTARY DATA [file supp_gku1172_nar-02021-z-2014-File012.zip › manual/javadoc/RNAstructure_java_drawing/source/proxy/StructureBackend.html]

StructureBackend


JavaScript is disabled on your browser.


- Overview
- Package
- Class
- Tree
- Index
- Help

- Prev Class
- Next Class

- Frames
- No Frames

- All Classes

- Summary:
- Nested |
- Field |
- Constr |
- Method

- Detail:
- Field |
- Constr |
- Method


RNAstructure\_java\_drawing.source.proxy

## Class StructureBackend

- java.lang.Object
- - RNAstructure\_java\_drawing.source.proxy.StructureBackend

- ---

    

  ```
  public class StructureBackend
  extends Object
  ```

- - ### Field Summary

    Fields

    | Modifier and Type | Field and Description |
    | --- | --- |
    | `protected boolean` | `swigCMemOwn` |
    | `private long` | `swigCPtr` |
  - ### Constructor Summary

    Constructors

    | Modifier | Constructor and Description |
    | --- | --- |
    |  | `StructureBackend()` |
    | `protected` | `StructureBackend(long cPtr, boolean cMemoryOwn)` |
  - ### Method Summary

    Methods

    | Modifier and Type | Method and Description |
    | --- | --- |
    | `boolean` | `addAnnotationProbability(String file)` |
    | `boolean` | `addAnnotationSHAPE(String file)` |
    | `void` | `delete()` |
    | `protected void` | `finalize()` |
    | `void` | `flip()` |
    | `protected static long` | `getCPtr(StructureBackend obj)` |
    | `String` | `getStructureData(int number)` |
    | `boolean` | `readStructureData(String file)` |
    | `void` | `removeAnnotation()` |
    | `void` | `setNucleotidesCircled(boolean circled)` |
    | `String` | `writeDotBracketFile(String inFile, String outFile)` |
    | `String` | `writeHelixFile(String inFile, String outFile, int number)` |
    | `void` | `writePostscriptFile(String outFile, int number)` |
    | `void` | `writeSVGFile(String outFile, int number)` |

    - ### Methods inherited from class java.lang.Object

      `clone, equals, getClass, hashCode, notify, notifyAll, toString, wait, wait, wait`

- - ### Field Detail


    - #### swigCMemOwn

      ```
      protected boolean swigCMemOwn
      ```


    - #### swigCPtr

      ```
      private long swigCPtr
      ```
  - ### Constructor Detail


    - #### StructureBackend

      ```
      public StructureBackend()
      ```


    - #### StructureBackend

      ```
      protected StructureBackend(long cPtr,
                      boolean cMemoryOwn)
      ```
  - ### Method Detail


    - #### addAnnotationProbability

      ```
      public boolean addAnnotationProbability(String file)
      ```


    - #### addAnnotationSHAPE

      ```
      public boolean addAnnotationSHAPE(String file)
      ```


    - #### delete

      ```
      public void delete()
      ```


    - #### finalize

      ```
      protected void finalize()
      ```

      **Overrides:**
      :   `finalize` in class `Object`


    - #### flip

      ```
      public void flip()
      ```


    - #### getCPtr

      ```
      protected static long getCPtr(StructureBackend obj)
      ```


    - #### getStructureData

      ```
      public String getStructureData(int number)
      ```


    - #### readStructureData

      ```
      public boolean readStructureData(String file)
      ```


    - #### removeAnnotation

      ```
      public void removeAnnotation()
      ```


    - #### setNucleotidesCircled

      ```
      public void setNucleotidesCircled(boolean circled)
      ```


    - #### writeDotBracketFile

      ```
      public String writeDotBracketFile(String inFile,
                               String outFile)
      ```


    - #### writeHelixFile

      ```
      public String writeHelixFile(String inFile,
                          String outFile,
                          int number)
      ```


    - #### writePostscriptFile

      ```
      public void writePostscriptFile(String outFile,
                             int number)
      ```


    - #### writeSVGFile

      ```
      public void writeSVGFile(String outFile,
                      int number)
      ```


- Overview
- Package
- Class
- Tree
- Index
- Help

- Prev Class
- Next Class

- Frames
- No Frames

- All Classes

- Summary:
- Nested |
- Field |
- Constr |
- Method

- Detail:
- Field |
- Constr |
- Method
